# *Bulbophyllum pingnanense* (Orchidaceae, Epidendroideae, Dendrobiinae), a new species from Fujian, China

**DOI:** 10.3897/phytokeys.65.8254

**Published:** 2016-06-30

**Authors:** Jiang-Feng Liu, Si-Ren Lan, Bi-Zhu He, Yi-Chi Liang

**Affiliations:** 1 College of Landscape Architecture, Fujian Agriculture and Forestry University, Fuzhou 350002, China; 2 College of Horticulture, Fujian Agriculture and Forestry University, Fuzhou 350002, China; 3 Management Office of Yushan Scenic Area, Fuzhou350001, China

**Keywords:** Bulbophyllum, Eastern China, Fujian, Orchidaceae

## Abstract

A new orchid species, *Bulbophyllum pingnanense*, is described and illustrated from Fujian, China. It is similar to *Bulbophyllum brevipedunculatum* and *Bulbophyllum albociliatum* in vegetative and floral morphology, but it can be distinguished from *Bulbophyllum brevipedunculatum* by having a longer dorsal sepal with longer white ciliate on margin, longer and lanceolate lateral sepals, and a glabrous lip. It can be distinguished from *Bulbophyllum albociliatum* by having a shorter inflorescence, and a longer dorsal sepal.

## Introduction

*Bulbophyllum* Thouars is one of the largest orchid genera. It includes more than 1900 species and extends widely from tropical America, Africa, Madagascar, and mainland Asia to Australasia (Lindley 1830, Pearce and Cribb 2002, Seidenfaden 1979, 1992, Chen et al. 2009, Pridgeon et al. 2014). There are about 105 species of *Bulbophyllum* in China, according to the most recent revision and recently published new species (Chen et al. 2009, Jin et al. 2014, Hu et al. 2015). Section *Cirrhopetalum* (Lindley) Reichenbach is characterized by sub-umbellate inflorescence, shorter dorsal than lateral sepals, twisting and connected lateral sepals, and hairy dorsal sepal and petals (Seidenfaden 1979). *Cirrhopetalum* includes 57 species, 17 (10 endemic) of which are found in China (Chen et al. 2009). During fieldwork in Pingnan County, northeastern Fujian, a new species of *Bulbophyllum*, best placed under the section *Cirrhopetalum*, was found and described below.

## Materials and methods

Gross morphological data were obtained during fieldwork. Measurements, shapes, colours and other details given in the description were based on living material. The images of flowering plant were photographed with the Canon S100v digital camera. The floral anatomy was conducted under a XTL-340Z stereomicroscope.

## Taxonomy

### Bulbophyllum pingnanense

Taxon classificationPlantaeAsparagalesOrchidaceae

J.F. Liu, S.R. Lan & Y.C. Liang sp. nov.

urn:lsid:ipni.org:names:77155742-1

Figs 1 , 2

#### Type.

China. Fujian: Pingnan County, Shuangxi Town, on rock along Yuanyan River, 800–900 m, 27°01'N, 119°05'E, 23 June 2013, J.F. Liu 201312 (holotype: FAFU!; isotype: NOCC!).

#### Diagnosis.

*Bulbophyllum pingnanense* is similar to *Bulbophyllum brevipedunculatum* T.C. Hsu & S.W. Chung and *Bulbophyllum albociliatum* (T.S. Liu & H.Y. Su) K. Nackejima. It differs from *Bulbophyllum brevipedunculatum* by having a longer dorsal sepal with either an obtuse or an acute apex and longer white ciliate on margins; longer and lanceolate lateral sepals; and glabrous lip. It can be distinguished from *Bulbophyllum albociliatum* by its shorter inflorescence, a longer dorsal sepal with either an obtuse or an acute apex.

#### Description.

Epiphytic herb. Rhizome creeping, slender, 0.6–1 mm in diam. Pseudobulbs 0.6–2.5 cm apart on rhizome, obovate-elliptic, 0.5–1.7 cm, 3–6 mm in diam., with a terminal leaf. Leaf sessile; blade oblong to linear-oblong, 1.8–6.6 × 0.6–1.2 cm, apex obtuse to retuse. Scape arising from base of pseudobulb, ca. 1.1 cm, umbel 3–5 flowered; peduncle slender, ca. 0.6 mm in diam., with 3 or 4 sheaths; floral bracts triangular, 2–3 mm. Pedicel and ovary ca. 4 mm. Flowers orange red. Dorsal sepal concave, ovate, abaxially papillose, ca. 5 × 3 mm, margins long white ciliate, apex obtuse or acute; lateral sepals lanceolate, abaxially papillose, 10–12 × ca. 2 mm, slightly twisted near the base, with their upper and lower edges often loosely adhering, margins glabrous, apex acute. Petals ovate, 2.7–3.0 × 1.2–2.0 mm, margins long white ciliate, apex rounded. Lip recurved, ovate-triangular, ca. 3 mm, abaxially deeply grooved, base attached to end of column foot by a mobile joint. Column yellow, subterete, ca. 1–2 mm, with a distinct foot, ca. 1.0–2.5 mm, conspicuously winged; stelidia triangular, slender; anther cap subglobose; pollinia 4, in 2 pairs, without appendages.

**Figure 1.**
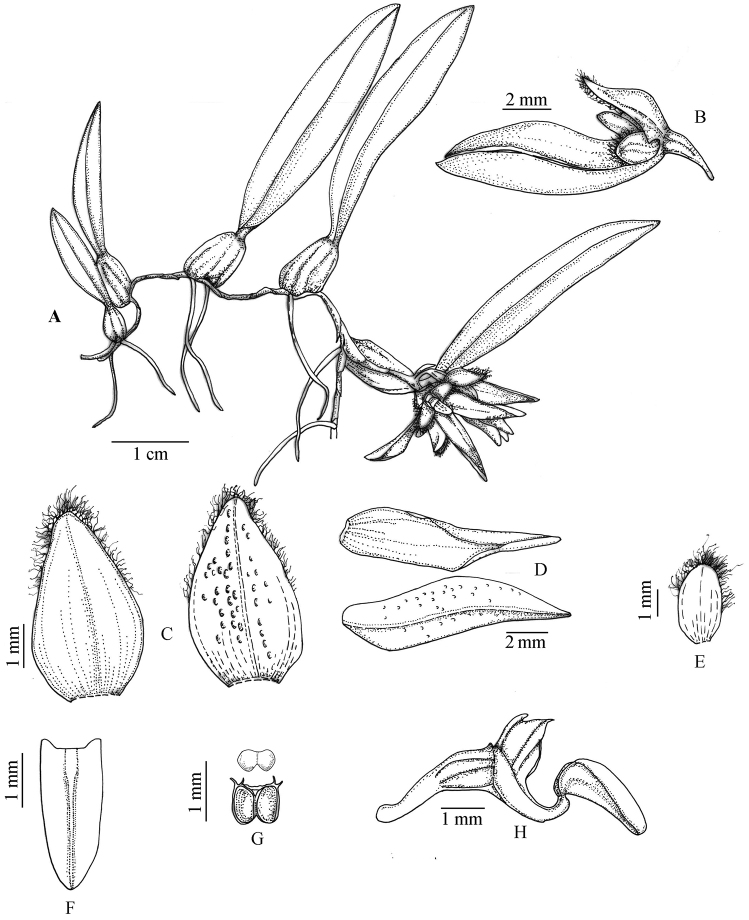
*Bulbophyllum pingnanense* sp. nov. **A** plant **B** flower **C** dorsal sepal **D** lateral sepal **E** petal **F** lip **G** pollinia and anther cap **H** lip, column, pedicel and ovary, side view (Drawn from the holotype by Bi-Dan Lai).

**Figure 2.**
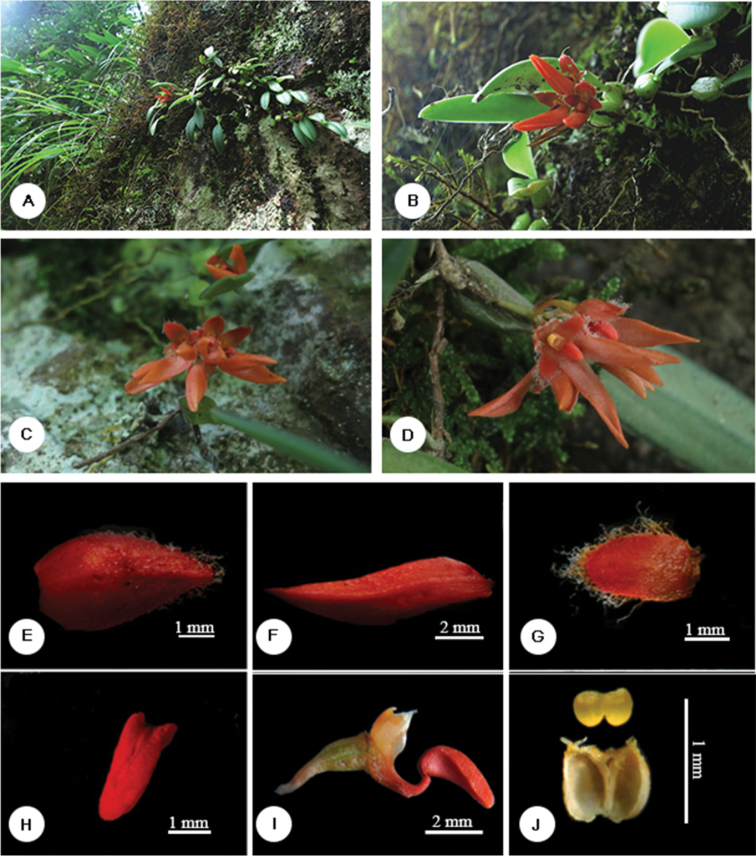
*Bulbophyllum pingnanense* J.F. Liu, S.R. Lan & Y.C. Liang. **A** habitat and habit **B–D** flower **E** dorsal sepal **F** lateral sepal **G** petal **H** lip **I** lip, column, pedicel and ovary, side view **J** pollinia and anther cap.

#### Distribution and habitat.

**Figure 3.**
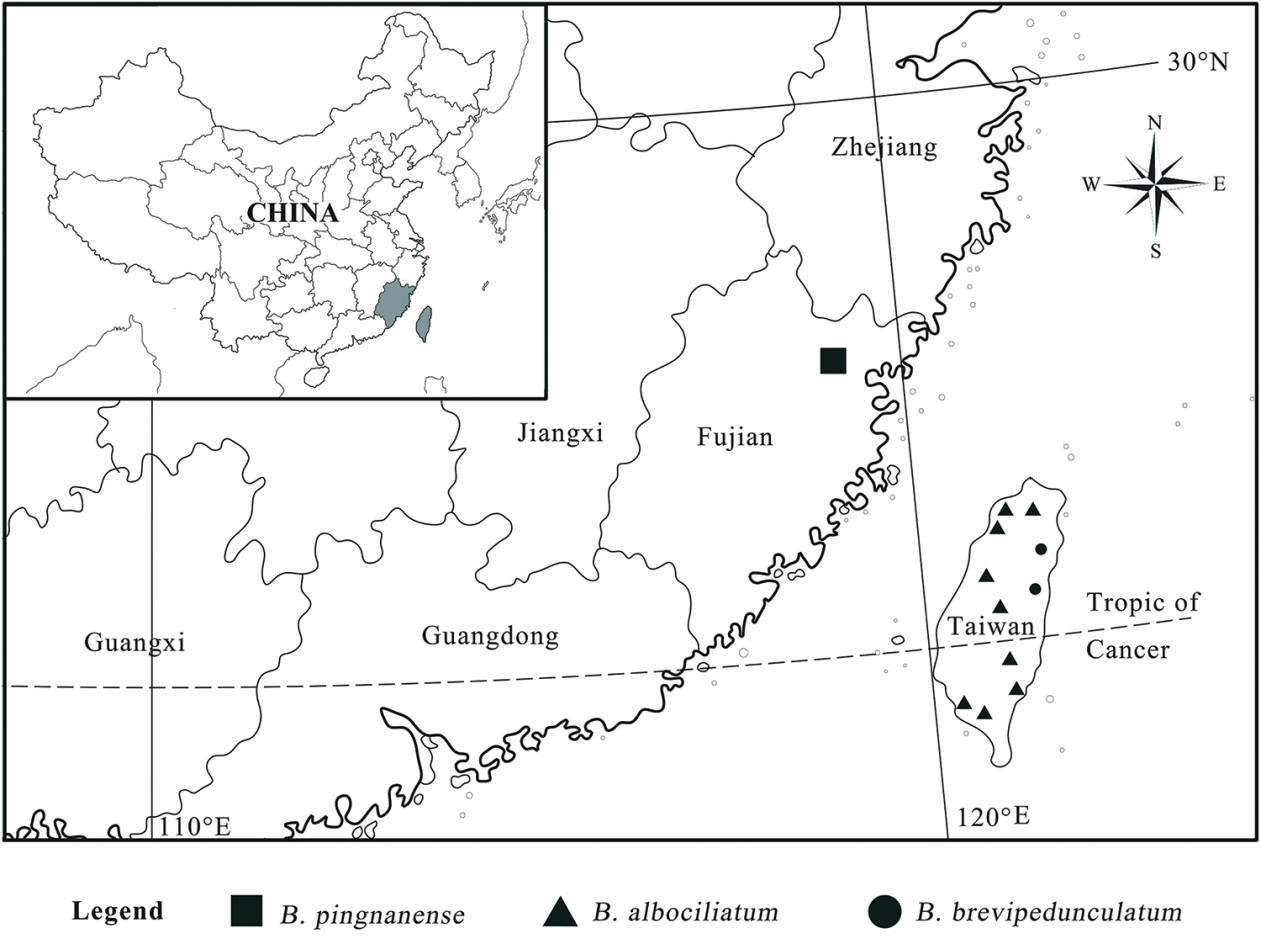
Distribution of *Bulbophyllum pingnanense*, *Bulbophyllum brevipedunculatum* and *Bulbophyllum albociliatum*.

*Bulbophyllum pingnanense* is so far only known within Pingnan, Fujian, China (Fig. 3). It is epiphytic on steep rock in the edge of evergreen coniferous and broad-leaved mixed forest, which is mainly composed of *Castanopsis eyrei* (Champ. ex Benth.) Hutch. (Fagaceae), *Cunninghamia lanceolata* (Lamb.) Hook. (Taxodiaceae). Other orchids, *Amitostigma gracile* (Bl.) Schltr., *Pholidota cantonensis* Rolfe, *Cymbidium floribundum* Lindl. and *Pleione formosana* Hayata, were found growing nearby this new species.

#### Phenology.

Flowering from June to July.

#### Conservation status.

*Bulbophyllum pingnanense* is known only from the type locality, and only one population of ca. 3000 individual plants was discovered in a small area of ca. 0.002 km^2^ during two years of botanical surveys. Based on the extent of occurrence estimated to be less than 100 km^2^ (CR B1) and the area of occupancy less than 10 km^2^ (CR B2), species existing at a single location (CR B1a + B2a), *Bulbophyllum pingnanense* is assigned a preliminary status of Critically Endangered (CR B1a + B2a) according to the IUCN Categories and Criteria (IUCN 2012). In addition, the plants of *Bulbophyllum* are used as herbal medicine in the locality. It is possible that *Bulbophyllum pingnanense* might also be collected for using as herbal medicine. Therefore, immediate conservation strategy should be taken.

#### Etymology.

The species epithet refers to Pingnan County where this new species was found.

#### Taxonomic notes.

Several morphological characters, such as the dorsal sepal being much shorter than the lateral sepals, and the lateral sepals twisting and connected, indicate that this species belongs to sect. *Cirrhopetalum*. *Bulbophyllum pingnanense* is closely related to *Bulbophyllum brevipedunculatum*, but it can be distinguished by having a longer (ca. 5 mm vs. ca. 3.5 mm) dorsal sepal with either an obtuse or an acute (vs. rounded) apex and longer white ciliate (vs. short white ciliate) on margins, longer (10–12 mm vs. 5–7 mm) and lanceolate (vs. near rectangular) lateral sepals, glabrous (vs. adaxially papillose) lip. In addition, the two species had different flowering seasons that never overlapped (June–July vs. March–April), and the nearest distance between them is ca. 370 km, separated by the sea (Fig. 3). The new species is also closely related to *Bulbophyllum albociliatum*, but it can be distinguished by having a shorter (ca. 1.1 cm vs. 4–6 cm) inflorescence, and a longer (ca. 5 mm vs. 3–4 mm) dorsal sepal with either an obtuse or an acute (vs. rounded) apex; the nearest distance between the two species is ca. 350 km, separated by the sea (Fig. 3).

### Key to the related species of *Bulbophyllum pingnanense*

**Table:** 

1	Scape ca. as long as pseudobulb	**2**
–	Scape much longer than pseudobulb	**4**
2	Lateral sepals near rectangular, 5–7 mm long, ca. 2 × as long as dorsal sepal or shorter	***Bulbophyllum brevipedunculatum***
–	Lateral sepals narrowly oblong or lanceolate, ca. 10 mm long, ca. 2 × as long as dorsal sepal or longer	**3**
3	Scape ca. 4 mm, sepals yellow, lip triangular-lanceolate	***Bulbophyllum henanense***
–	Scape ca. 11 mm, sepals orange red, lip ovate-triangular	***Bulbophyllum pingnanense***
4	Adaxial surface of lip glabrous, lateral sepals 0.7–1.1 cm	***Bulbophyllum albociliatum***
–	Adaxial surface of lip partly papillose, lateral sepals 1.2–1.4 cm	***Bulbophyllum kuanwuense***

## Supplementary Material

XML Treatment for Bulbophyllum pingnanense
